# Publisher Correction: NRG1 type I dependent autoparacrine stimulation of Schwann cells in onion bulbs of peripheral neuropathies

**DOI:** 10.1038/s41467-019-09886-4

**Published:** 2019-04-16

**Authors:** Robert Fledrich, Dagmar Akkermann, Vlad Schütza, Tamer A. Abdelaal, Doris Hermes, Erik Schäffner, M. Clara Soto-Bernardini, Tilmann Götze, Axel Klink, Kathrin Kusch, Martin Krueger, Theresa Kungl, Clara Frydrychowicz, Wiebke Möbius, Wolfgang Brück, Wolf C. Mueller, Ingo Bechmann, Michael W. Sereda, Markus H. Schwab, Klaus-Armin Nave, Ruth M. Stassart

**Affiliations:** 1 Institute of Anatomy, University of Leipzig, Liebigstr. 13, 04103 Leipzig, Germany; 2 Department of Neurogenetics, Max-Planck-Institute of Experimental Medicine, Hermann-Rein-Str. 3, 37075 Göttingen, Germany; 3 Department of Neuropathology, University Hospital Leipzig, Liebigstr. 26, 04103 Leipzig, Germany; 4 Department of Clinical Neurophysiology, University Medical Center Göttingen, Robert-Koch-Str. 40, 37075 Göttingen, Germany; 5 Center for Research in Biotechnology (CIB), Costa Rican Institute of Technology (TEC), Cartago, Costa Rica; 6 Department of Cellular Neurophysiology, Hanover Medical School, Carl-Neuberg-Str. 1, 30625 Hanover, Germany; 7 Center Nanoscale Microscopy and Molecular Physiology of the Brain (CNMPB), Göttingen, Germany; 8 Institute of Neuropathology, University Medical Center Göttingen, Robert-Koch-Str. 40, 37075 Göttingen, Germany; 9 Center for Systems Neuroscience (ZSN), Bünteweg 2, 30559 Hanover, Germany

Correction to: *Nature Communications* 10.1038/s41467-019-09385-6, published online 01 April 2019

The original version of this Article contained errors in the author affiliations.

Michael W. Sereda was incorrectly associated with the Department of Cellular Neurophysiology, Hanover Medical School, Carl-Neuberg-Str. 1, 30625 Hanover, Germany. The correct affiliations for Michael W. Sereda are Department of Neurogenetics, Max-Planck-Institute of Experimental Medicine, Hermann-Rein-Str. 3, 37075 Göttingen, Germany and Department of Clinical Neurophysiology, University Medical Center Göttingen, Robert-Koch-Str. 40, 37075 Göttingen, Germany.

Markus H. Schwab was incorrectly associated with Department of Clinical Neurophysiology, University Medical Center Göttingen, Robert-Koch-Str. 40, 37075, Göttingen. The correct affiliations for Markus H. Schwab are Department of Neurogenetics, Max-Planck-Institute of Experimental Medicine, Hermann-Rein-Str. 3, 37075 Göttingen, Germany; Department of Cellular Neurophysiology, Hanover Medical School, Carl-Neuberg-Str. 1, 30625 Hanover, Germany; and Center for Systems Neuroscience (ZSN), Bünteweg 2, 30559 Hanover, Germany.

Ruth M. Stassart was incorrectly associated with the Center for Research in Biotechnology (CIB), Costa Rican Institute of Technology (TEC), Cartago, Costa Rica. The correct affiliations for Ruth M. Stassart are Department of Neurogenetics, Max-Planck-Institute of Experimental Medicine, Hermann-Rein-Str. 3, 37075 Göttingen, Germany and Department of Neuropathology, University Hospital Leipzig, Liebigstr. 26, 04103 Leipzig, Germany.

These errors have now been corrected in both the PDF and HTML versions of the Article.

