# Supplementary material for: NRG1 type I dependent autoparacrine stimulation of Schwann cells in onion bulbs of peripheral neuropathies
Source: Nat Commun. 2019 Apr 1;10:1467. doi: 10.1038/s41467-019-09385-6 (PMC6443727; doi:10.1038/s41467-019-09385-6)
Supplement: Supplementary file 1 — Supplementary information [file 41467_2019_9385_MOESM1_ESM.pdf]

Supplementary Information

NRG1 type I dependent autoparacrine stimulation of Schwann cells in onion bulbs of peripheral neuropathies

by Fledrich and Akkermann *et al.*

Supplementary Figure 1

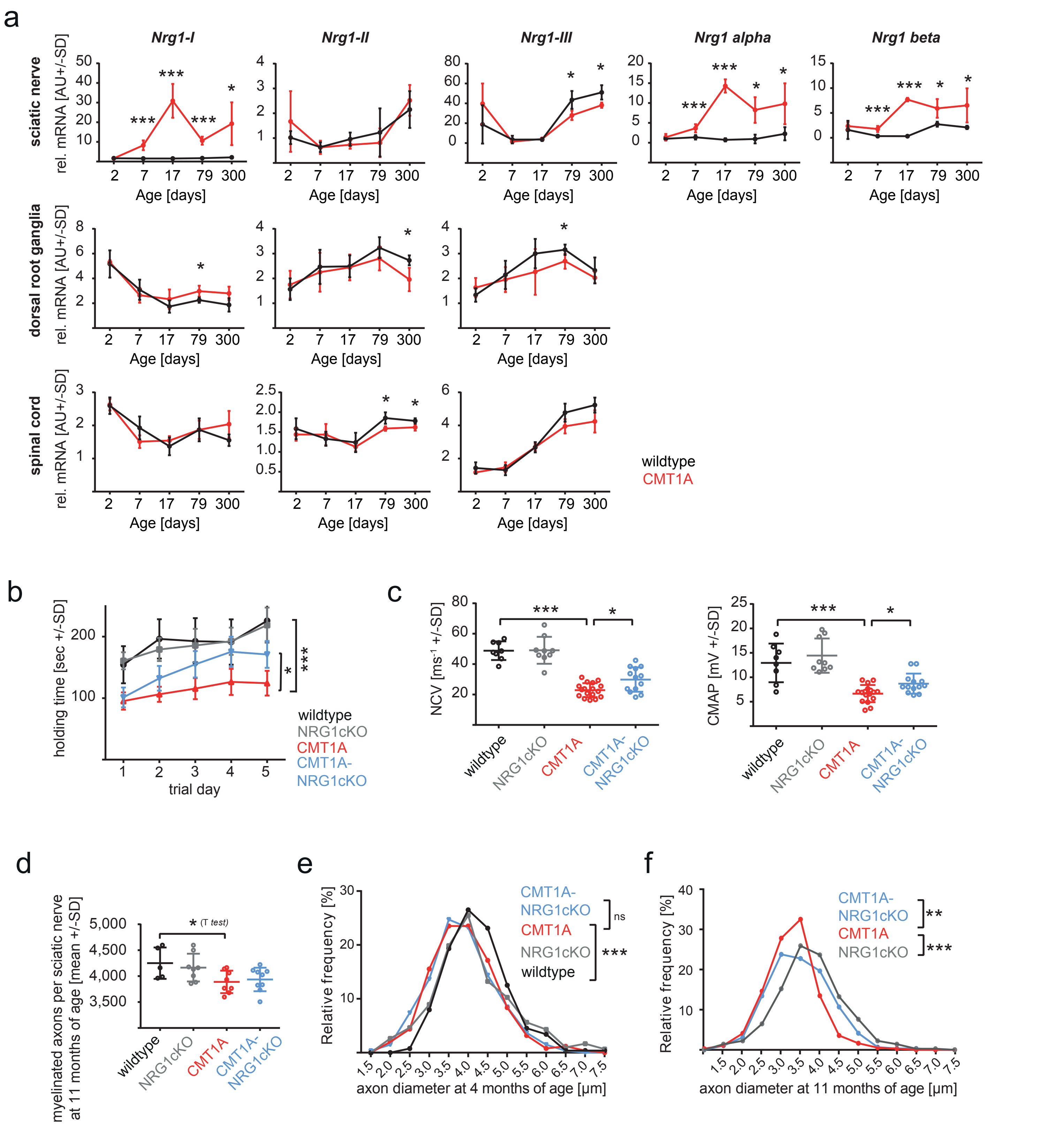

Supplementary figure 1:

(**a**) Relative mRNA expression of different *Nrg1* isoforms in sciatic nerve (top row), dorsal root ganglia (middle row) and spinal cord (lower row) comparing CMT1A (red) and wildtype (black) mice at the age of 2, 7, 17, 79 and 300 days. Note that only *Nrg1-I* and both, the alpha and beta isoforms, are induced in sciatic nerves of CMT1A mice (n=3-6 per group and time point, multiple Student's T tests).

(**b**) Accelerating rotarod analysis of 11 months old mice on five consecutive days reveals a reduced holding time in CMT1A mice that is rescued by deletion of glial *Nrg1* in CMT1A animals (wildtype n=4, NRG1cKO n=8, CMT1A n=8, CMT1A-NRG1cKO n=11, two-way ANOVA with Tukey's multiple comparison tests).

(**c**) Quantification of nerve conduction velocity (NCV, left panel) and the compound muscle action potential (CMAP, right panel) in 11 months old wildtype (n=8), NRG1cKO (n=9), CMT1A (n=17) and CMT1A-NRG1cKO (n=13) mice. NCV and CMAP are improved in CMT1A-NRG1cKO mice compared to CMT1A animals whereas sole glial Nrg1 knock out (NRG1cKO) does not alter both parameters compared to wildtype (one-way ANOVA with Tukey's post test).

(**d**) Lightmicroscopic quantification of the number of myelinated axons per sciatic nerve cross section at the age of 11 months revealed reduction in CMT1A (n=8) compared to wildtype (n=5) mice, but no alteration by ablation of glial *Nrg1* in CMT1A (CMT1A-NRG1cKO, n=9) when compared to single CMT1A mice (one-way ANOVA, Tukey's post test).

(**e**) At four months of age, the axonal caliber, as extracted from the g-ratio measurements in **Fig.2h** is decreased in CMT1A mice (red) when compared to wildtype controls (black). However, no rescue in axonal caliber is observed upon ablation of Nrg1 from Schwann cells in CMT1A mice (blue, wildtype n=5, NRG1cKO n=5, CMT1A n=5, CMT1A-NRG1cKO n=5, 220-375 fibers were measured per animal, multiple Kolmogorov-Smirnov tests).

(**f**) At eleven months of age, the axonal caliber is still decreased in CMT1A mice (red; n=6) when compared to controls (grey; NRG1cKO n=5). However, now axonal caliber is improved upon ablation of *Nrg1* from Schwann cells in CMT1A mice (blue; CMT1A-NRG1cKO, n=4; 221-363 fibers were measured per animal, multiple Kolmogorov-Smirnov tests).

Supplementary Figure 2

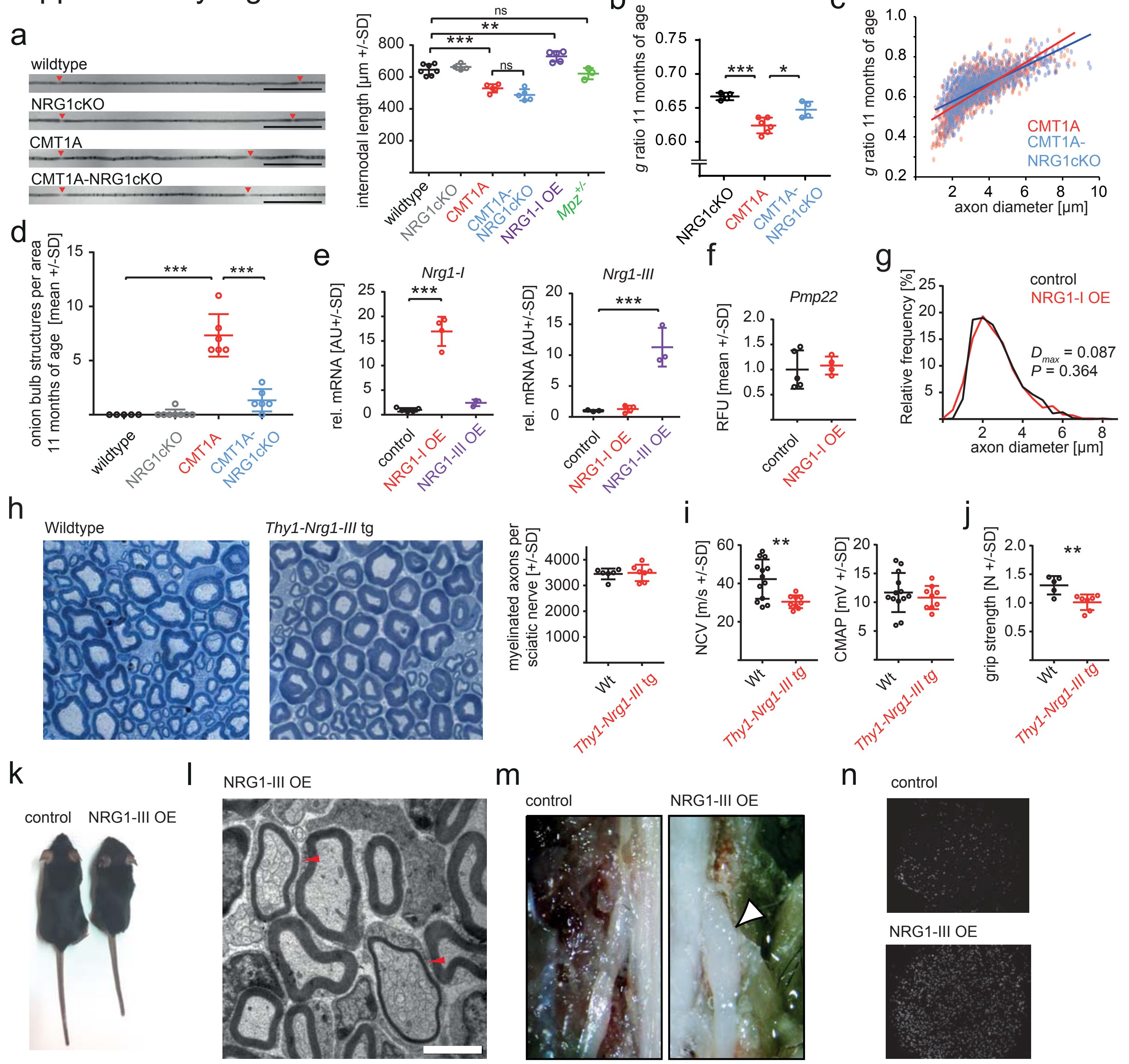

Supplementary figure 2:

(**a**) Representative images (left panels) of teased fibers from tibial nerves of four months old mice and respective quantification (right panel) showing shortened internodal length in *PMP22* tg mice compared to wildtypes and controls (NRG1cKO). No alteration in internodal length is found upon ablation of *Nrg1* in Schwann cells of CMT1A animals compared to single CMT1A mice (wildtype n=4, NRG1cKO n=4, CMT1A n=5, CMT1A-NRG1cKO n=5, NRG1-I OE n=5, Mpz+/- n=3, one-way ANOVA and Tukey's post test, scale bar: 200 µm).

(**b**) Quantification of the mean myelin sheath thickness (*g*-ratio) at the age of 11 months revealed an amelioration of the decreased g-ratio in CMT1A mice after ablation of Schwann cell *Nrg1* (NRG1cKO n=5, CMT1A n=6, CMT1A-NRG1cKO n=4, 221-363 fibers were measured per animal, one-way ANOVA with Tukey's post test).

(**c**) Scatterblots of quantified myelin sheath thickness (*g*-ratio, data of **b**) plotted against the axon diameter shows hypermyelination of small to mid-caliber and hypomyelination of large caliber fibers in CMT1A mice (red). The regression line in CMT1A-NRG1cKO mice (blue) compared to CMT1A indicates ameliorated hypermyelination of small to mid-caliber axons in CMT1A mice with ablated Nrg1 in Schwann cells.

(**d**) Electronmicroscopic quantification of onion bulb structures per area (15,740µm²) in cross sections of sciatic nerve of 11 months old mice (wildtype n=5, NRG1cKO n=8, CMT1A n=6, CMT1A-NRG1cKO n=6) showing the reduction of onion bulbs upon glial ablation of *Nrg1* in CMT1A mice (one-way ANOVA, Tukey's post test).

(**e**) Relative mRNA expression of *Nrg1-I* (left) and *Nrg1-III* (right) in sciatic nerve of two months old mice either overexpressing *Nrg1-I* (NRG1-I OE, red) or *Nrg1-III* (NRG1-III OE, purple) in Schwann cells (relative to *Rplp0* and *Ppia*; ctrl, n=3-5; NRG1-I OE, n=4; NRG1-III OE, n=3; one-way ANOVA and Tukey's post test).

(**f**) Relative *Pmp22* mRNA expression in four months old mice shows no difference upon overexpression of glial *Nrg1-I* (NRG1-I OE, n=5) compared to controls (n=4, Student's T test).

(**g**) At four months of age, the axonal caliber, as extracted from the g-ratio measurements in **Fig.3h** is unaltered in NRG1-I OE mice (n=4, red) when compared to controls (n=5, black, Kolmogorov-Smirnov test).

(**h**) Representative light microscopic images (left and middle panel) of sciatic nerve cross sections of 12 months old wildtype and *Thy1-Nrg1-III* tg mice and respective quantification (right panel) of myelinated axons per sciatic nerve cross section shows no difference between the groups (wt n=6, *Thy1-Nrg1-III* tg n=7, Student's T test).

(**i**) Analyses of the nerve conduction velocity (NCV) and the compound muscle action potential (CMAP) in 12 months old wildtype and *Nrg1-III* tg mice shows a decrease in NCV upon neuronal *Nrg1-III* overexpression and no alteration of the CMAP (wt n=13, *Thy1-Nrg1-III* tg n=9, Student´s T test).

(**j**) Grip strength test of hind limbs in 12 months old animals shows a decrease in grip strength upon axonal *Nrg1-III* overexpression (wt n=5, *Thy1-Nrg1-III* tg n=7, Student´s T test).

(**k**) Representative picture of a two months old mice overexpressing *Nrg1-III* in Schwann cells (NRG1-III OE, right) and a litter mate control (left) emphasizing the growth retardation of mutant animals.

(**l**) Electron micrograph of a sciatic nerve cross section from a two months old NRG1-III OE mouse. Arrows indicate aberrant Remak bundle ensheathment (scale bar 2.5 µm)

(**m**) Photographs of exposed spinal cord with nerve roots (from ventral) from two months old mice showing a massive hyperplasia of the nerve root (indicated by arrow head) in a mouse overexpressing *Nrg1-III* (right) compared to control (left).

(**n**) Sciatic nerve cross sections of two months old mice showing massive increase of nuclei (DAPI, white) in a mouse overexpressing *Nrg1-III* in Schwann cells (lower panel) compared to a control (upper panel).

Supplementary Figure 3

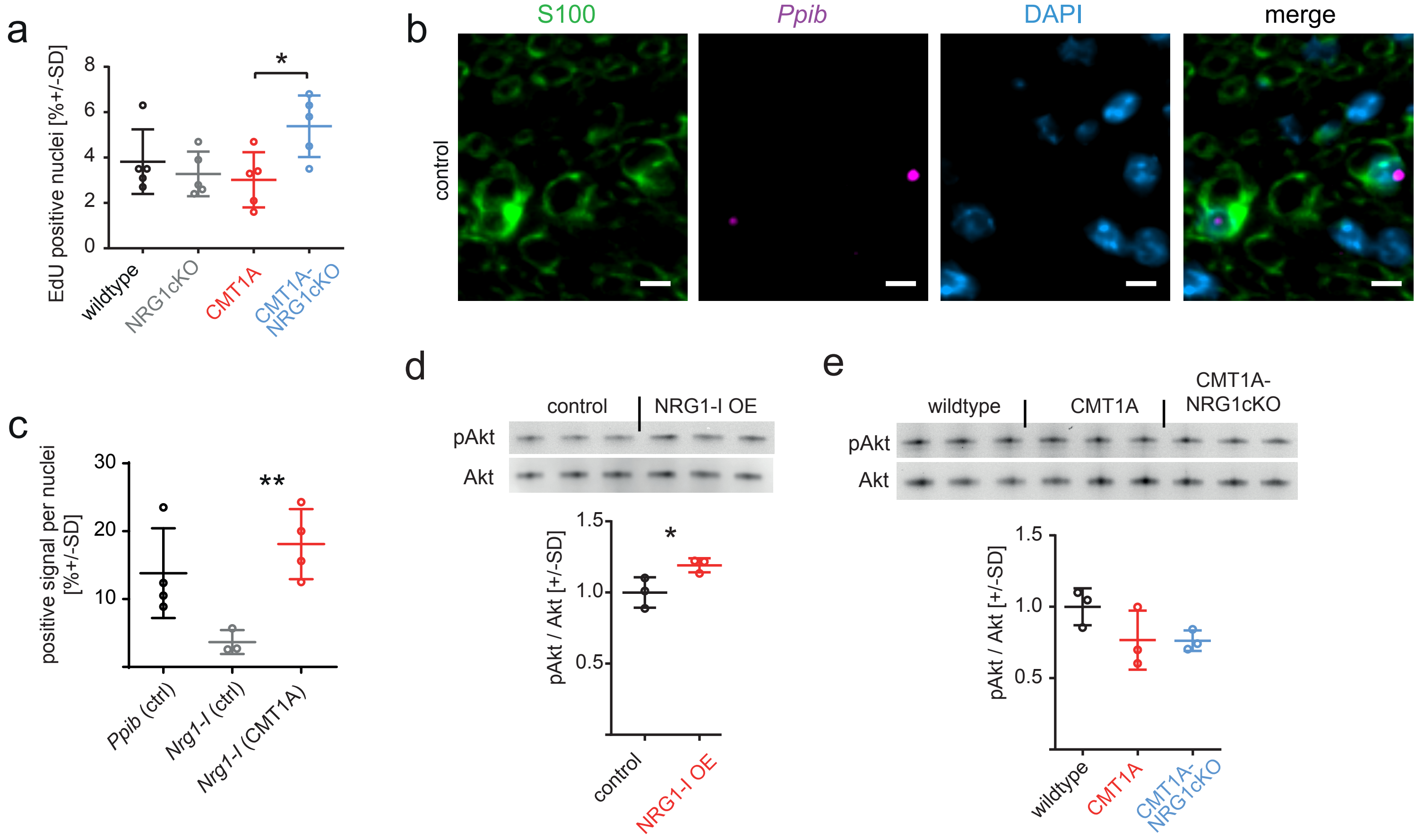

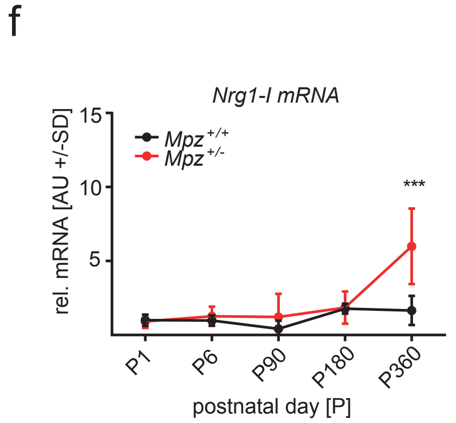

Supplementary figure 3:

(**a**) Analysis of Schwann cell proliferation *in vivo* by quantification of EdU positive nuclei per sciatic nerve cross section at P11 (EdU injection at P10) in wildtype (black, n=5), NRG1cKO (grey, n=5), CMT1A (red, n=5) and CMT1A-NRG1cKO (blue, n=5) mice (one-way ANOVA and Tukey's post test).
(**b**) BaseScope *in situ* hybridization in Schwann cells (S100, green) in sciatic nerve cross section of a four month old wildtype mouse against the positive control probe *Ppib* (magenta, nuclei in blue).
(**c**) Quantification of BaseScope *in situ* hybridization in sciatic nerve cross sections of four month old control (black and grey, n=3-4) and CMT1A mice (red, n=4) for either the housekeeping probe *Ppib* (from **b**) or the target mRNA *Nrg1-I*. Note that the percentage of positive nuclei is comparable between the positive control (*Ppib*) in wildtype and *Nrg1-I* in mutant nerves (one-way ANOVA and Tukey's post test).
(**d**) Western blot analyses (upper panel) and respective quantifications (lower panel) of sciatic nerve lysates of four months old mice showing an increased phosphorylation of Akt in NRG1-I OE mice compared to controls (n=3 per group, Student's T test).
(**e**) Western blot analyses (left panels) and respective quantifications (right panel) of sciatic nerve lysates of four months old mice showing no improvement of Akt phosphorylation upon glial Nrg1 ablation in CMT1A mice (n=3 per group).
(**f**) The mRNA expression of *Nrg1-I* in sciatic nerves of Mpz+/- mice (red), a model for CMT1B, is induced only in later disease stages after P180 when compared to controls (Mpz+/+, black, n=3-5 per timepoint, multiple Student's T tests).

Supplementary Figure 4

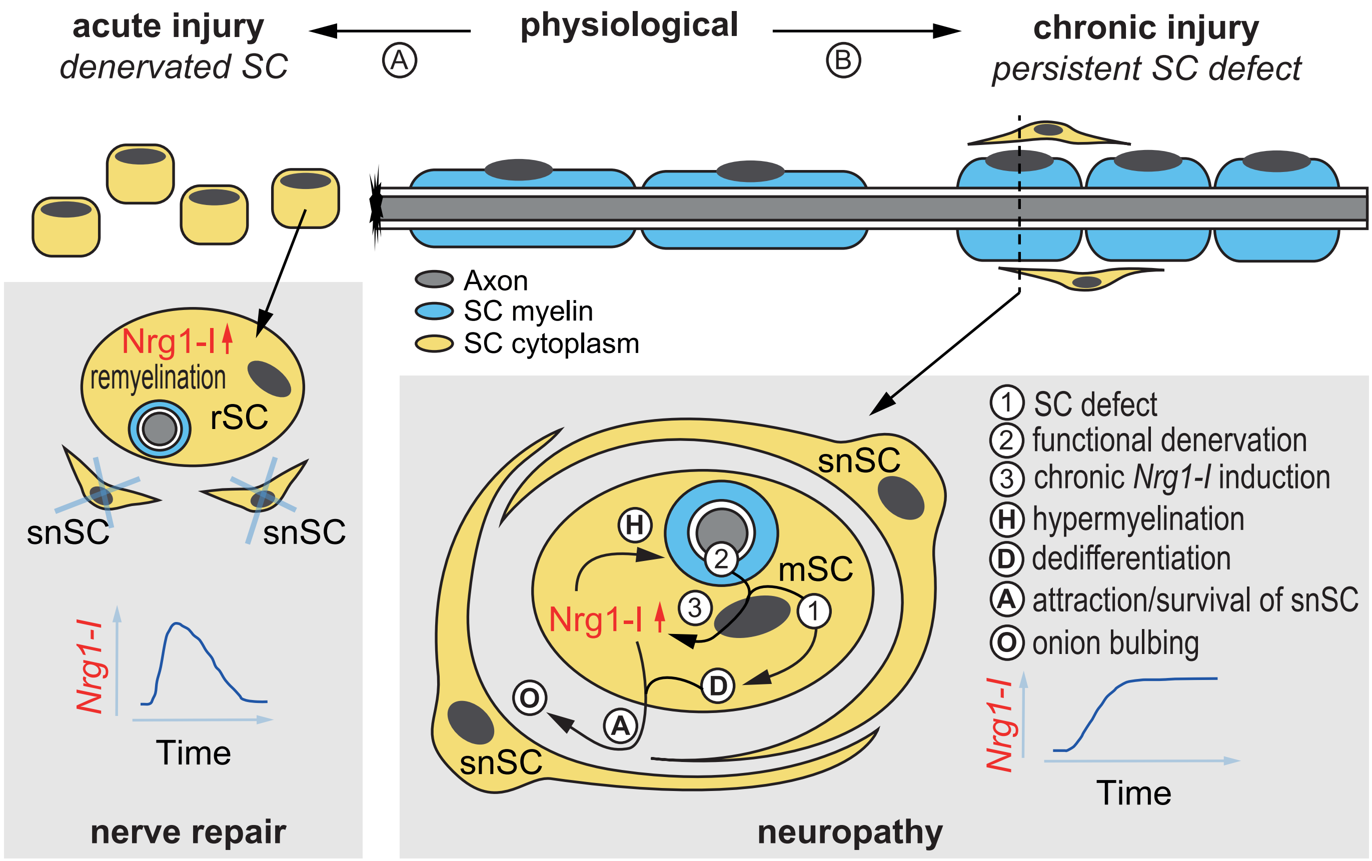

Hypothetical model of the role of Schwann cell NRG1-I in acute and chronic nerve injury. After acute nerve injury (left panel, A), denervated Schwann cells (dSC) transiently express *NRG1-I* which supports redifferentiation and remyelination[29]. In contrast to acute injury, NRG1-I is continuously expressed by myelinating Schwann cells that are characterized by a permanent defect in chronic CMT1A disease (right panel, B). In detail, a persistent primary defect (1) in myelinating Schwann cells (mSC) in CMT1A (caused by *PMP22* overexpression) leads to functional denervation, characterized by an impaired access to (and/or integration of) axonal NRG1 signaling cues (2). This triggers the induction of soluble *NRG1-I* expression in Schwann cells (3). Autocrine NRG1-I subsequently induces hypermyelination (H) of small caliber axons. On the other hand, dedifferentiation (D) of myelinating (mSC) and supernumerary Schwann cells (snSC) together with paracrine NRG1-I signaling promotes the survival and attraction (A) of supernumerary Schwann cells (snSC), ultimately leading to the formation of onion bulb structures (O). While transient glial *NRG1-I* upregulation after injury supports nerve repair, the persistent *NRG1-I* expression in demyelinating neuropathies causes a detrimental disease response which impairs nerve function and the clinical phenotype in a model for CMT1A disease.

Supplementary Figure 5: Full length Western blots

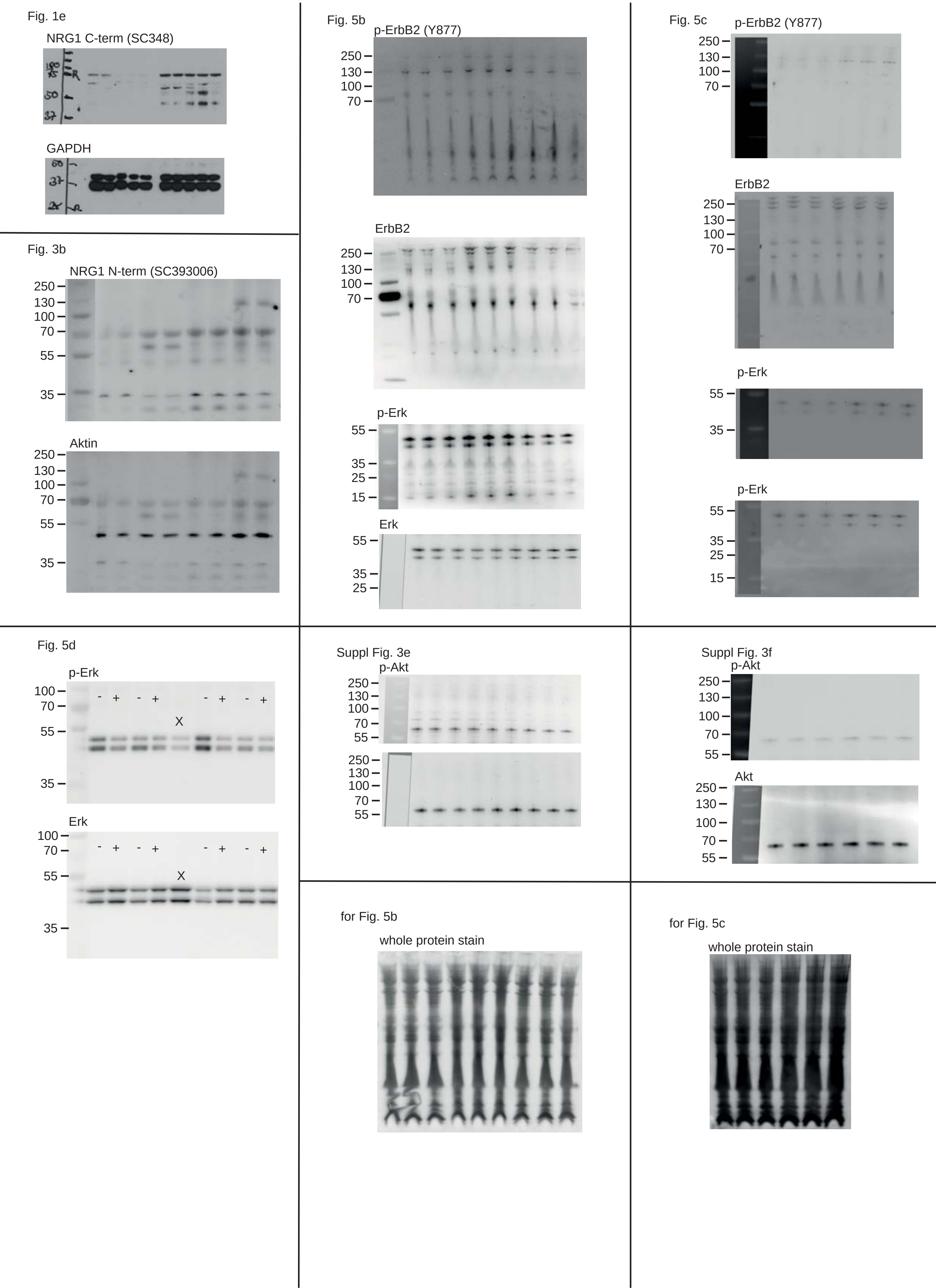

Supplementary table 1: primer sequences

Gene	species	primer	sequence
Nrg1-I	mouse	sense	5'-GGGAAGGGCAAGAAGAAGG -3'
		antisense	5'-TTTCACACCGAAGCACGAGC -3'
Nrg1-I	rat	sense	5'-GGGAAGGGCAAGAAGAAGG -3'
		antisense	5'-TTTCGCACCGGAGCACTAGC -3'
NRG1-I	human	sense	5'-GGGAAGGGCAAGAAGAAGG -3'
		antisense	5'-TTTCACACCGAAGGACTAGT -3'
Nrg1-I 5'UTR	mouse	sense	5'-TTTCTGCAAGCCCTTGGACC -3'
		antisense	5'-TTTCACACCGAAGCACGAGC -3'
Nrg1-II	mouse	sense	5'-CCTCGTTTCCTCCGCTG -3'
		antisense	5'-TTTCACACCGAAGCACGAGC -3'
Nrg1-III	mouse	sense	5'-ACTCAGCCACAAACAACAGAAAC -3'
		antisense	5'-GAAGCACTCGCCTCCATT -3'
Nrg1 alpha	mouse	sense	5'-GTGTGCGGAGAAGGAGAAAACT -3'
		antisense	5'-TCTTGCTCCAGTGAATCCAGGTTG -3'
Nrg1 beta	mouse	sense	5'-GTGTGCGGAGAAGGAGAAAACT -3'
		antisense	5'-TGGCAACGATCACCAGTAAACTCA -3'
Rplp0	mouse	sense	5'-TTCGGAGTTTCTTGTGATCTTCC -3'
		antisense	5'-ACAATGAAGCATTTTGGATAATCA -3'
Rplp0	rat	sense	5'-GATGCCCAGGGAAGACAG -3'
		antisense	5'-CACAATGAAGCATTTTGGGTAG -3'
Ppia	mouse	sense	5'-CACAAACGGTTCCCAGTTTT -3'
		antisense	5'-TTCCCAAAGACCACATGCTT -3'
Ppia	rat	sense	5'-AGCACTGGGGAGAAAGGATT -3'
		antisense	5'-AGCCACTCAGTCTTGGCAGT -3'
S100	human	sense	5'-GAGCTTTCCCATTTCTTAGAGGA-3'
		antisense	5'-GAAGTCACATTCGCCGTCTC-3'
Pou3f1	mouse/rat	sense	5'-GCGTGTCTGGTTCTGCAAC -3'
		antisense	5'-AGGCGCATAAACGTCGTC -3'
cJun	mouse/rat	sense	5'-CCTTCTACGACGATGCCCTC -3'
		antisense	5'-GGTTCAAGGTCATGCTCTGTTT -3'
Sox2	mouse	sense	5'-TCCAAAAACTAATCACAACAATCG -3'
		antisense	5'-GAAGTGCAATTGGGATGAAAA -3'
Ngfr	mouse	sense	5'-CGGTGTGCGAGGACACTGAGC -3'
		antisense	5'-TGGGTGCTGGGTGTTGTGACG -3'
Olig1	mouse	sense	5'-CCGCCCCAGATGTACTATGC -3'
		antisense	5'-AACCCACCAGCTCATACAGC -3'
Shh	mouse	sense	5'-AAAGCTGACCCCTTTAGCCTA -3'
		antisense	5'-TTCGGAGTTTCTTGTGATCTTCC -3'
Pmp22	mouse/rat	sense	5'-AATGGACACACGACTGATC -3'
		antisense	5'-CCTTTGGTGAGAGTGAAGAG -3'
