# Supplementary material for: NRG1 type I dependent autoparacrine stimulation of Schwann cells in onion bulbs of peripheral neuropathies
Source: Nat Commun. 2019 Apr 1;10:1467. doi: 10.1038/s41467-019-09385-6 (PMC6443727; doi:10.1038/s41467-019-09385-6)
Supplement: Supplementary file 3 — Reporting Summary [file 41467_2019_9385_MOESM3_ESM.pdf]

nature research

Corresponding author(s): Dr. Ruth M.Stassart, Dr. Robert Fledrich, Prof. Klaus-Armin Nave, Dr. Markus Schwab

Last updated by author(s): Feb 18, 2019

Reporting Summary

Nature Research wishes to improve the reproducibility of the work that we publish. This form provides structure for consistency and transparency in reporting. For further information on Nature Research policies, see Authors & Referees and the Editorial Policy Checklist.

Statistics

For all statistical analyses, confirm that the following items are present in the figure legend, table legend, main text, or Methods section.

n/a	Confirmed	
☐	☒	The exact sample size (*n*) for each experimental group/condition, given as a discrete number and unit of measurement
☐	☒	A statement on whether measurements were taken from distinct samples or whether the same sample was measured repeatedly
☐	☒	The statistical test(s) used AND whether they are one- or two-sided<br>*Only common tests should be described solely by name; describe more complex techniques in the Methods section.*
☐	☒	A description of all covariates tested
☐	☒	A description of any assumptions or corrections, such as tests of normality and adjustment for multiple comparisons
☐	☒	A full description of the statistical parameters including central tendency (e.g. means) or other basic estimates (e.g. regression coefficient) AND variation (e.g. standard deviation) or associated estimates of uncertainty (e.g. confidence intervals)
☐	☒	For null hypothesis testing, the test statistic (e.g. *F*, *t*, *r*) with confidence intervals, effect sizes, degrees of freedom and *P* value noted<br>*Give P values as exact values whenever suitable.*
☒	☐	For Bayesian analysis, information on the choice of priors and Markov chain Monte Carlo settings
☒	☐	For hierarchical and complex designs, identification of the appropriate level for tests and full reporting of outcomes
☒	☐	Estimates of effect sizes (e.g. Cohen's *d*, Pearson's *r*), indicating how they were calculated

Our web collection on statistics for biologists contains articles on many of the points above.

Software and code

Policy information about availability of computer code

Data collection	not applicable
Data analysis	not applicable

For manuscripts utilizing custom algorithms or software that are central to the research but not yet described in published literature, software must be made available to editors/reviewers. We strongly encourage code deposition in a community repository (e.g. GitHub). See the Nature Research guidelines for submitting code & software for further information.

Data

Policy information about availability of data

All manuscripts must include a data availability statement. This statement should provide the following information, where applicable:

- Accession codes, unique identifiers, or web links for publicly available datasets
- A list of figures that have associated raw data
- A description of any restrictions on data availability

All data supporting the findings of this manuscript are available upon reasonable request from the corresponding authors.

Field-specific reporting

Please select the one below that is the best fit for your research. If you are not sure, read the appropriate sections before making your selection.

☒ Life sciences ☐ Behavioural & social sciences ☐ Ecological, evolutionary & environmental sciences

For a reference copy of the document with all sections, see nature.com/documents/nr-reporting-summary-flat.pdf

Life sciences study design

All studies must disclose on these points even when the disclosure is negative.

Sample size	Wherever applicable, sample sizes were predetermined using the software G*Power3.1.9.2 (http://www.gpower.hhu.de/). Default settings were ANOVA tests (F tests) with alpha error of 0.05 and beta error of 0.2. Whenever previous comparable data sets were available, the effect sizes were calculated with known means and variances to finally estimate the required sample sizes.
Data exclusions	No data was excluded.
Replication	Replication in vitro was performed at least three times with independent cell culture preparations. Replication in vivo was performed by biological replicates.
Randomization	For animal studies, organisms were allocated randomly to the experimental groups with only considering the determined genotypes.
Blinding	Animal experiments (phenotype analyses, electrophysiology and histology) were conducted in a single blinded fashion towards the investigator. Selection of animal samples out of different experimental groups for molecular biology/histology/biochemistry was performed randomly and in a blinded fashion.

Reporting for specific materials, systems and methods

We require information from authors about some types of materials, experimental systems and methods used in many studies. Here, indicate whether each material, system or method listed is relevant to your study. If you are not sure if a list item applies to your research, read the appropriate section before selecting a response.

Materials & experimental systems

n/a	Involved in the study	
☐	☒	Antibodies
☒	☐	Eukaryotic cell lines
☒	☐	Palaeontology
☐	☒	Animals and other organisms
☐	☒	Human research participants
☒	☐	Clinical data

Methods

n/a	Involved in the study	
☒	☐	ChIP-seq
☒	☐	Flow cytometry
☒	☐	MRI-based neuroimaging

Antibodies

Antibodies used	NRG1 (C-terminal, pRb, Santa Cruz, #SC348), cJUN (mRb, Cell Signaling, #9165), MPZ (mM, kind gift of JJ Archelos), p-AKT (Ser473, pRb, Cell Signalling, #3787), AKT (pRb, Cell Signalling, #9275), p-ERK1/2 (Thr202/Tyr204, pRb, Cell Signalling, #9101), ERK 1/2 (pRb, Cell Signalling, #4695), p-ERBB2 (Y877, pRb, Cell Signaling, cat.#2241), ERBB2 (pRb, Santa Cruz, sc-284), GAPDH (mM, Enzo Life Sciences, Clone 1D4, ADI-CSA-335-E), Actin (mM, EMD Millipore, Clone C4, MAB1501), NRG1-N-terminal (mM, Santa Cruz, #SC393006)
Validation	NRG1 antibodies were validated by Western blotting with tissue from NRG1 gain and loss of function mouse mutants. P-Akt/Akt, P-Erk/Erk and P-ErbB2/ErbB2 antibodies were validated by Western blotting with protein extracts from primary Schwann cells that treated with or without recombinant NRG1. MPZ and cJUN were validated by ilocalization in mmunhistochemistry of healthy and acutely injured nerves in mice.

Animals and other organisms

Policy information about studies involving animals; ARRIVE guidelines recommended for reporting animal research

Laboratory animals	C57/Bl6 wildtype animals, Pmp22 transgenic rats Tg(Pmp22)Kan)1, PMP22 transgenic mice (Tg(PMP22)C61Clh)2, Pmp22+/- mice (Pmp22tm1Ueli)3, Mpz-/- mice (Mpztm1Msch)4, Mpz+/-mice (Mpztm1Msch)5, mice transgenic for neuronal Nrg1 type I (C57BL/6-Tg(Thy1-Nrg1*I)1Kan+/–, Thy1-Nrg1(I) tg) and neuronal Nrg1 type III (C57BL/6-Tg(Thy1-Nrg1*III)1Kan+/–, Thy1-Nrg1(III) tg)6, the Dhh-Cre driver line (FVB(Cg)-Tg(Dhh-cre)1Mejr/J; DhhCre)7, Nrg1-flox (Nrg1tm3CBm; Nrg1fl/fl)8 and SodG93A mice (B6SJL-Tg(SOD1*G93A)1Gur/J)9, DhhCrexNrg1-/ß1a and Nrg1-IIIß1a stop flox mice
Wild animals	Not applicable
Field-collected samples	Not applicable
Ethics oversight	All experiments within this manuscript are approved by the ethic board of the University Medical Center Göttingen (human data) and the Niedersächsische Landesamt für Verbraucherschutz und Lebensmittelsicherheit (LAVES, animal data).

Note that full information on the approval of the study protocol must also be provided in the manuscript.

Human research participants

Policy information about studies involving human research participants

Population characteristics	Selection of patients was performed upon availability of fresh frozen material and according to exclusion/inclusion criteria. Samples were anonymized and processed in a blinded manner. Selected patients were of mixed age, between >30 years and <70 years of age (inclusion criteria) and did not suffer from another severe neurological disorder at time-point of biopsy (exclusion criteria)
Recruitment	Selection of patients was performed upon availability of fresh frozen material.
Ethics oversight	All patients did provide informed consent in accordance with the ethical compliance guidelines. The study received ethical approval by the ethic board of the University Medical Center Göttingen, Germany.

Note that full information on the approval of the study protocol must also be provided in the manuscript.
